# TACT: Research Partner involvement in a cancer clinical trials unit and academic research centre; evaluating its impact

**DOI:** 10.1186/1745-6215-16-S1-P13

**Published:** 2015-05-29

**Authors:** Noreen Hopewell-Kelly, Jim Fitzgibbon, Jessica Baillie, Annmarie Nelson

**Affiliations:** 1 Marie Curie palliative Care research Centre, Cardiff University, Cardiff, Wales, UK; 2 School of Health Care Sciences, Cardiff University, Cardiff, Wales, UK

## Background

Since 2005 lay representatives (‘Research Partners’ – RP) have been involved in the work of a clinical trials unit and academic research centre. Their roles can include attending trial management groups, reviewing documents and chairing and presenting at sub-committees. Where recruitment of RPs was once opportunistic, RPs are now more formally recruited in conjunction with a national public involvement organisation.

The impact of RPs at the research centre had not been examined in-depth, nor research partners’ or staff members’ experiences been previously explored. The TACT study was conducted to investigate the input and impact of RPs to ensure the best possible working partnership between the centre and the public is achieved.

## Method

Semi-structured interviews were conducted with RPs (n= 10) and staff members (n=8). The data were analysed using a Framework approach.

## Results

Research partners and members of staff see the RP role as an advocacy role for patients. Although some RPs feel welcomed into the centre and are happy with their level of involvement, others identify more negative points including an apparent bias in the centre’s tendency to use more experienced RPs, the RP role being a funding requirement that is tokenistic in its implementation and the need for greater monitoring and support within the RP role.

Staff members stated that they were unclear about the degree to which RPs should be involved in their work and the processes involved in working alongside RPs. Although there was a general recognition that greater commitment was required of them in the RP initiative, time pressures and stresses were cited as barriers in achieving this aim.

## Conclusion

The evaluation demonstrated that the RP role is generally valued and enjoyed by RPs but this is only theoretically reciprocated by members of staff who face challenges and barriers in fully committing to the RP initiative.

